# Long-Term Results of a Propensity Score Analysis Comparing 5 × 5 Gy and 10 × 3 Gy of Radiotherapy Alone for Malignant Spinal Cord Compression

**DOI:** 10.3390/jcm14248741

**Published:** 2025-12-10

**Authors:** Dirk Rades, Darejan Lomidze, Carlos Ferrer-Albiach, Antonio Jose Conde-Moreno, Barbara Segedin, Blaz Groselj, Raquel Ciérvide Jurio, Fernando López Campos, Charlotte Kristiansen, Kristopher Dennis, Jon Cacicedo

**Affiliations:** 1 Department of Radiation Oncology, University Medical Center Schleswig-Holstein, Lübeck Campus, 23538 Lübeck, Germany; 2 Department of Radiation Oncology, University of Lübeck, 23562 Lübeck, Germany; 3 Radiation Oncology Department, Tbilisi State Medical University, Tbilisi 0177, Georgia; dlomidze@hotmail.com; 4 Radiation Oncology Department, Ingorokva High Medical Technology University Clinic, Tbilisi 0177, Georgia; 5 Department of Radiation Oncology, Consorcio Hospital Provincial de Castellon, 12002 Castellon, Spain; carlos.ferrer@hospitalprovincial.es (C.F.-A.); conde_ant@gva.es (A.J.C.-M.); 6 Department of Radiation Oncology, University and Polytechnic Hospital La Fe, 46026 Valencia, Spain; 7 Department of Radiotherapy, Institute of Oncology Ljubljana, 1000 Ljubljana, Slovenia; bsegedin@onko-i.si (B.S.); bgroselj@onko-i.si (B.G.); 8 Department of Radiotherapy, Faculty of Medicine, University of Ljubljana, 1000 Ljubljana, Slovenia; 9 Department of Radiation Oncology, University Hospital HM Sanchinarro, 28050 Madrid, Spain; rciervide@hmhospitales.com; 10 Department of Radiation Oncology, University Hospital Ramón y Cajal, 28034 Madrid, Spain; flcampos@salud.madrid.org; 11 Department of Oncology, Vejle Hospital, University Hospital of Southern Denmark, 7100 Vejle, Denmark; charlotte.kristiansen@rsyd.dk; 12 Division of Radiation Oncology, The Ottawa Hospital and the University of Ottawa, Ottawa, ON K1Y 4E9, Canada; 13 Radiation Oncology Department, Hospital Universitario Cruces, 48903 Barakaldo, Spain; jon.cacicedofernandezbobadilla@osakidetza.eus; 14 Radiation Oncology Department, Biobizkaia Health Research Institute, 48903 Barakaldo, Spain

**Keywords:** malignant spinal cord compression, radiation therapy alone, dose-fractionation, overall treatment time, long-term outcomes

## Abstract

**Background/Objectives:** For many patients with malignant spinal cord compression (MSCC) not suitable for surgery, irradiation alone is the preferred treatment. A dose-fractionation regimen of 10 × 3 Gy is common for this situation. Since most patients suffer from motor deficits and pain, the number of radiotherapy sessions should be as low as possible. A secondary analysis of a phase 2 trial compared 5 × 5 Gy to a historical control group treated with 10 × 3 Gy. After 1:2 matching, 5  ×  5 Gy appeared similarly effective regarding local progression-free survival (LPFS) at 6 months, motor function, walking ability, and overall survival. **Methods:** This retrospective study investigated whether these findings are consistent in a larger cohort and after longer follow-up. Additional data were gathered for the phase 2 cohort, follow-up in the control group was not limited to 6 months, and the number of patients in the control group increased from 213 to 728. **Results:** After propensity-score matching, no significant differences were found regarding LPFS after 12 (*p* = 0.198), 18 (*p* = 0.139), and 24 (*p* = 0.117) months, effect on motor function (*p* = 0.393), walking ability (*p* = 0.079), 24-month local control (*p* = 0.655), and 24-month OS (*p* = 0.403). **Conclusions:** Given the limitations of this study, 5 × 5 Gy appears preferable to 10 × 3 Gy in selected patients receiving irradiation without upfront surgery. Clinicians should balance initial convenience for the patients and future limitations when re-irradiation in the same part of the spine is required.

## 1. Introduction

Malignant spinal cord compression (MSCC) was reported to develop in up to 10% of cancer patients during the course of their malignant disease [1,2]. If patients are not suitable for initial neurosurgery or do not wish to receive such an intervention, irradiation alone, complemented by concurrent administration of dexamethasone if safely possible, is the treatment of choice for the majority of this patient group [1,2]. For patients selected for irradiation without surgery, several dose-fractionation regimens are applied worldwide [1,2]. A common regimen includes 10 fractions of 3.0 Gy (10 × 3 Gy) over two weeks, i.e., administered over ten consecutive working days. However, because most patients with MSCC suffer from motor deficits and vertebral pain, the number of radiotherapy sessions should be kept as low as reasonably possible [1,2]. Therefore, a prospective phase 2 trial (PRE-MODE) was conducted between 2017 and 2018, which investigated the potential of 5 × 5 Gy over one week [3]. In a secondary analysis of this trial, patients receiving 5 × 5 Gy were matched and compared to patients irradiated with 10 × 3 Gy [4]. As the equivalent dose in 2-Gy fractions (EQD2) for tumor cell kill, which considers both the total dose and the dose per fraction, of 5 × 5 Gy is very similar to the EQD2 of 10 × 3 Gy (31.3 Gy vs. 32.5 Gy), comparable outcomes of both regimens were expected [5]. And indeed, 5 × 5 Gy appeared similarly effective as 10 × 3 Gy in terms of the local progression-free survival (LPFS) at 6 months, effect of irradiation on motor function, post-treatment walking ability, and 6-month overall survival (OS) [4]. However, the follow-up period in the phase 2 trial was limited to 6 months, and the follow-up in the historical control group was limited to 6 months [4]. Thus, it remained unclear whether the findings of that study would be consistent after a longer period of follow-up. Moreover, due to the procedure of 1:2 matching, the total number of patients in the previous study was limited to 96, i.e., 32 patients receiving 5 × 5 Gy and 64 patients receiving 10 × 3 Gy [4]. Therefore, this additional study was performed including additional data for the phase 2 cohort, a longer follow-up in the historical control group, and a much larger sample size in the control group. In this study, propensity score methods were performed for the comparison of 5 × 5 Gy and 10 × 3 Gy. In comparison to the previous study, the follow-up period could now be extended up to 24 months following irradiation.

## 2. Materials and Methods

This study was approved by the Ethics Committee at the University of Lübeck (2025-403). It evaluated long-term outcomes of 40 patients previously included in a prospective phase 2 trial (PRE-MODE, clinicaltrials.gov NCT0307043, registered 3 March 2017). The patients were treated with 5 × 5 Gy of volumetric modulated arc therapy or stereotactic body radiation therapy administered over one week for motor deficits caused by MSCC. The treatment was performed at university hospitals (*N* = 39) or a provincial hospital (N = 1) between 02/2017 and 03/2018 [3]. The data for this prospective cohort were compared to the outcomes of a historical control group consisting of 728 patients who received 10 × 3 Gy of conventional irradiation over one week between 1992 and 2022 and met the same eligibility criteria as required for inclusion in the PRE-MODE trial. These criteria included MSCC in the thoracic or lumbar parts of the spine, motor deficits of the legs present for a maximum of 30 days, confirmation of MSCC by magnetic resonance imaging or computed tomography, no neurologic deficits due to other reasons than MSCC, and no clear indication for upfront surgery [3]. Concurrent administration of dexamethasone was strongly recommended. Patients with previous irradiation or spinal surgery in the areas of MSCC or involvement of only the cervical spine were not considered. The baseline characteristics of both patient groups are shown in Table 1.

For the comparisons between both dose groups, propensity score methods were performed to reduce the risk of selection biases caused by differences regarding baseline characteristics [6]. These methods considered ten characteristics (Table 1), i.e., age, gender, primary tumor, period between cancer diagnosis and MSCC, number of affected spinal segments (vertebrae), other distant metastases (bone or other organs), dynamic (time) of motor deficits, walking ability, and performance score according to the Eastern Cooperative Oncology Group (ECOG) [7]. Based on propensity scores, patients were assigned to one of five strata, given by the quintiles of the propensity scores. To see whether selection biases were removed successfully, Cochran–Mantel–Haenszel tests were applied. For all of these statistical analyses, SAS software (v9.4; SAS, Cary, NC, USA) was used. For additional information regarding materials and methods, please refer to the publication of the original PRE-MODE trial [4].

The primary goal of the present additional study was the evaluation of long-term results beyond 6 months. To achieve this goal, additional data were gathered from patients of the prospective trial who were alive at 6 months following irradiation, and the follow-up in the control group was not limited to 6 months. Moreover, to increase the number of patients and the statistical power in the present study, the control group was considerably expanded from 213 to 728 patients [4]. LPFS (at least no further progression of motor deficits during the course of irradiation and no in-field recurrence of MSCC afterwards) up to 24 months is the main endpoint of the present study. Additional endpoints include the effect of irradiation on motor deficits (improvement vs. no further progression vs. deterioration), walking ability after treatment (able to walk with or without aid vs. unable to walk), local control (freedom from an in-field recurrence of MSCC, assessed in patients with improvement or no further progression of motor deficits during the course of irradiation), and overall survival (OS). For grading of motor deficits of the legs, a 5-point scale was used (0 = strength not reduced, 1 = patient is able to walk without aid, 2 = patient is able to walk with aid, 3 = patient is not able to walk, and 4 = paraplegia) [8].

## 3. Results

### 3.1. Descriptive Analyses

In the present study, additional follow-up data were obtained from 16 patients (40.0%) of the phase 2 cohort. Moreover, 281 patients (38.6%) in the historical control group had a follow-up period longer than 6 months. In the descriptive analyses comparing the phase 2 cohort and the control group (log-rank test), LPFS rates were 80.9% vs. 75.7% after 12 months (*p* = 0.090), 80.9% vs. 73.9% after 18 months (*p* = 0.161), and 80.9% vs. 66.0% after 24 months (*p* = 0.104), respectively. Local control rates were 85.1% vs. 89.9% (*p* = 0.622), 85.1% vs. 87.7% (*p* = 0.822), and 85.1% vs. 78.4% (*p* = 0.728), respectively. An in-field recurrence of MSCC in the previously irradiated part of the spine associated with motor deficits and requiring re-treatment occurred in two patients of the phase 2 cohort after 9 months and 9.5 months, respectively. In the control group, 35 patients developed an in-field recurrence leading to motor deficits and requiring re-treatment after a median of 8 months (range: 2–27 months). OS rates were 32.9% vs. 37.6% (*p* = 0.286), 29.9% vs. 31.7% (*p* = 0.111), and 26.6% vs. 24.8% (*p* = 0.187), respectively. Moreover, the improvement of motor function following irradiation occurred in 24 patients (60.0%) of the phase 2 cohort and in 196 patients (26.9%) of the control group, respectively (*p* = 0.001, Chi-square test). Following irradiation, 33 (82.5%) and 474 patients (65.1%), respectively, were able to walk (*p* = 0.024). Radiation-induced myelopathy was not reported in any patient of this study.

### 3.2. Propensity Score Analysis

The 40 patients of the phase 2 cohort and 722 of the historical control group qualified for the propensity score analysis. Six patients of the control group who received less than 80% of the planned total radiation dose were not considered for the propensity score approach. The distribution of the ten investigated baseline characteristics stratified by propensity score quintiles in the phase 2 cohort and the remaining 722 patients of the control group is given in Table 2. After propensity score matching, no significant differences were found between the phase 2 cohort and the control group with respect to LPFS, local control, and OS after 12, 18, and 24 months, respectively. The corresponding results are summarized in Table 3 (LPFS), Table 4 (local control), and Table 5 (OS). Moreover, the effect of irradiation on motor function (*p* = 0.393) and post-treatment ability to walk (*p* = 0.079) were not significantly different (Table 6).

## 4. Discussion

Patients with MSCC who are not candidates for neurosurgery followed by spinal irradiation are often assigned to irradiation alone. For these patients, a variety of dose-fractionation regimens are available [1,2]. Considering the symptom burden, a course of irradiation would ideally be both effective and short. Therefore, the prospective PRE-MODE trial evaluated the role of 5 × 5 Gy administered on five consecutive working days [3]. In previous studies, this regimen was shown to be superior to 5 × 4 Gy and similarly effective as 10 × 3 Gy over two weeks up to 6 months after irradiation [3,4]. The main goal of the present study was to investigate whether the similarity of the results for 5 × 5 Gy and 10 × 3 Gy still exists after 12, 18, and 24 months. Further endpoints included local control and OS. An additional goal was to compare 5 × 5 Gy and 10 × 3 Gy with respect to shorter-term effects, namely the impact on motor function and the ability to walk after irradiation, in a larger cohort as in a previous study [4]. In the present study, the historical control group included 728 patients compared to 213 patients in the previous study [4].

After propensity score matching, which was performed to reduce the risk of selection biases, no significant differences were found between the two dose groups with respect to LPFS, local control, and OS at 12, 18, and 24 months [6]. Moreover, the rates of improvement of motor function and post-treatment ability to walk were not significantly different. Thus, the conclusion of the previous study that 5 × 5 Gy appeared similarly effective as 10 × 3 Gy was confirmed in the present study [4]. Moreover, this study showed that the findings regarding LPFS and OS at 6 months in the previous study are still valid after a longer period of follow-up, namely at 12, 18, and 24 months. These results can be explained by the EQD2 for tumor cell kill (alpha/beta ratio of 10 Gy), which was similar for 5 × 5 Gy and 10 × 3 Gy, i.e., 31.3 Gy vs. 32.5 Gy [6]. The fact that radiation myelopathy was not reported can also be explained by the EQD2 (alpha/beta ratio of 2 Gy). The corresponding doses were 43.75 Gy and 37.5 Gy, respectively [9]. These doses are below the tolerance dose of the spinal cord of 45–50 Gy, which represents a risk of myelopathy of 0.03% to 0.2% [10,11]. Considering the similar effectiveness and the lower number of required treatment sessions, 5 × 5 Gy may be considered preferable to 10 × 3 Gy.

However, when planning to use 5 × 5 Gy instead of 10 × 3 Gy, one has to be aware of the fact that the EQD2 of 5 × 5 Gy with respect to radiation myelopathy is higher than the EQD2 of 10 × 3 Gy, which would be a disadvantage if re-irradiation of the same part of the spine due to MSCC or painful bone metastases. As stated above, the EQD2 was 43.75 Gy for 5 × 5 Gy compared to 37.5 Gy for 10 × 3 Gy [9]. According to Doi et al., the risk of myelopathy after re-irradiation for MSCC is low if the EQD2 for each course of radiotherapy is less than 51 Gy, the interval between both courses is at least 6 months, and the cumulative EQD2 of both courses is 75 Gy or less [12]. These aspects need to be considered and should be discussed with the patients considered for 5 × 5 Gy. Moreover, one should be aware of the limitations of the present study, which include the retrospective nature of the data obtained from the historical control group. Despite the application of propensity score methods, the retrospective nature might have led to hidden selection biases. Moreover, the additional data collected in the phase 2 cohort beyond 6 months were also retrospective in nature. In addition to hidden selection biases, one has to be aware of the fact that in-field recurrences might have been missed due to a lack of a structured follow-up performed in prospective trials. In both groups, spinal imaging during the retrospective phase of the study was only performed in case of recurrent motor deficits. Moreover, the findings of the present study likely cannot be generalized to other cohorts of patients with MSCC, e.g., patients receiving upfront spinal surgery, patients treated with stereotactic body radiation therapy, and patients with a favorable survival prognosis [13,14,15,16,17,18,19,20,21,22,23,24,25,26,27,28,29,30,31,32,33,34]. Patients with longer expected survival times assigned to irradiation alone were reported to benefit from longer-course regimens including higher total doses in terms of improved LPFS [33,34]. Thus, additional prospective studies are required that confirm the results of the present study and investigate the role of 5 × 5 Gy in other patient groups. Until these data are available, patients with more favorable OS prognoses should be preferably treated with longer-course regimens such as 10 × 3 Gy, 15 × 2.5 to 2.633 Gy, or 20 × 2 Gy [33,34]. The 5 × 5 Gy regimen appears to be a good option for patients with intermediate OS prognoses. The expected OS time can be estimated with a corresponding scoring instrument [35].

## 5. Conclusions

In patients with MSCC assigned to irradiation alone, 5 × 5 Gy resulted in similar LPFS after 12, 18, and 24 months when compared to 10 × 3 Gy. Moreover, 5 × 5 Gy was similarly effective with respect to local control, OS, improvement of motor function, and post-treatment ability to walk. Given the limitations of this study, 5 × 5 Gy appears preferable to 10 × 3 Gy for selected patients, particularly for patients with intermediate survival prognoses, since it is similarly effective but requires fewer treatment sessions. However, clinicians should balance initial convenience for the patients (fewer treatment sessions) and future limitations when a second course of radiotherapy in the same part of the spine is required.

## Figures and Tables

**Table 1:** Baseline characteristics of the phase 2 cohort (PRE-MODE trial, *n* = 40) receiving 5 × 5 Gy and the historical control group treated with 10 × 3 Gy (*n* = 728) without propensity score adjustment.

Characteristic	Phase 2 Cohort N Patients (%)	Control Group N Patients (%)	*p*-Value *
Age			0.108
<65 years	24 (60.0)	342 (47.0)	
≥65 years	16 (40.0)	386 (53.0)	
Gender			0.467
Female	14 (35.0)	297 (40.8)	
Male	26 (65.0)	431 (59.2)	
Interval tumor diagnosis to MSCC			0.870
≤15 months	22 (55.0)	410 (56.3)	
>15 months	18 (45.0)	318 (43.7)	
Distant metastases (additional bone)			0.214
No	9 (22.5)	232 (31.9)	
Yes	31 (77.5)	496 (68.1)	
Distant metastases (other organs)			0.051
No	14 (35.0)	370 (50.8)	
Yes	26 (65.0)	358 (49.2)	
Primary tumor			0.529
Breast cancer	7 (17.5)	161 (22.1)	
Prostate cancer	3 (7.5)	109 (15.0)	
Myeloma/lymphoma	5 (12.5)	64 (8.8)	
Lung cancer	12 (30.0)	169 (23.2)	
Other tumors	13 (32.5)	225 (30.9)	
Time developing motor deficits			0.283
1–7 days	14 (35.0)	255 (35.0)	
8–14 days	7 (17.5)	202 (27.7)	
>14 days	19 (47.5)	271 (37.2)	
Pre-radiotherapy walking ability			0.975
Not able to walk	16 (40.0)	293 (40.2)	
Able to walk	24 (60.0)	435 (59.8)	
Number of affected spinal segments			0.005
1–2	26 (65.0)	310 (42.6)	
≥3	14 (35.0)	418 (57.4)	
ECOG performance score			0.816
1–2	18 (45.0)	314 (43.1)	
3–4	22 (55.0)	414 (56.9)	

ECOG: Eastern Cooperative Oncology Group; MSCC: metastatic spinal cord compression. * The *p*-values were obtained with the Chi-square test.

**Table 2:** Baseline characteristics of the phase 2 cohort (PRE-MODE) and the control group based on propensity score quintiles.

	Quintile 1		Quintile 2		Quintile 3		Quintile 4		Quintile 5	
	Phase 2 (N = 7) *n* (%)	Control (N = 178) *n* (%)	Phase 2 (N = 9) *n* (%)	Control (N = 293) *n* (%)	Phase 2 (N = 8) *n* (%)	Control (N = 183) *n* (%)	Phase 2 (N = 8) *n* (%)	Control (N = 58) *n* (%)	Phase 2 (N = 8) *n* (%)	Control (N = 10) *n* (%)
**Age**										
<65 years	2 (28.6)	32 (18.0)	2 (22.2)	144 (49.1)	8 (100)	124 (67.8)	4 (50.0)	30 (51.7)	8 (100)	10 (100)
≥65 years	5 (71.4)	146 (82.0)	7 (77.8)	149 (50.9)	0 (0.0)	59 (32.2)	4 (50.0)	28 (48.3)	0 (0.0)	0 (0.0)
*p* = 0.782										
**Gender**										
Female	4 (57.1)	76 (42.7)	2 (22.2)	139 (47.4)	4 (50.0)	60 (32.8)	4 (50.0)	20 (34.5)	0 (0.0)	0 (0.0)
Male	3 (42.9)	102 (57.3)	7 (77.8)	154 (52.6)	4 (50.0)	123 (67.2)	4 (50.0)	38 (65.5)	8 (100)	10 (100)
*p* = 0.660										
**Interval from tumor diagnosis to MSCC**										
≤15 months	5 (71.4)	99 (55.6)	3 (33.3)	169 (57.7)	5 (62.5)	103 (56.3)	5 (62.5)	32 (55.2)	4 (50.0)	3 (30.0)
>15 months	2 (28.6)	79 (44.4)	6 (66.7)	124 (42.3)	3 (37.5)	80 (43.7)	3 (37.5)	26 (44.8)	4 (50.0)	7 (70.0)
*p* = 0.780										
**Distant metastases (additional bone)**										
No	4 (57.1)	65 (36.5)	4 (44.4)	89 (30.4)	1 (12.5)	71 (38.8)	0 (0.0)	1 (1.7)	0 (0.0)	0 (0.0)
Yes	3 (42.9)	113 (63.5)	5 (55.6)	204 (69.6)	7 (87.5)	112 (61.2)	8 (100)	57 (98.3)	8 (100)	10 (100)
*p* = 0.836										
**Distant metastases (other organs)**										
No	4 (57.1)	106 (59.6)	2 (22.2)	143 (48.8)	2 (25.0)	82 (44.8)	3 (37.5)	30 (51.7)	3 (37.5)	4 (40.0)
Yes	3 (42.9)	72 (40.4)	7 (77.8)	150 (51.2)	6 (75.0)	101 (55.2)	5 (62.5)	28 (48.3)	5 (62.5)	6 (60.0)
*p* = 0.082										
**Primary tumor**										
Breast cancer	2 (28.6)	39 (21.9)	2 (22.2)	87 (29.7)	1 (12.5)	26 (14.2)	2 (25.0)	8 (13.8)	0 (0.0)	0 (0.0)
Prostate cancer	1 (14.3)	69 (38.8)	2 (22.2)	21 (7.2)	0 (0.0)	12 (6.6)	0 (0.0)	3 (5.2)	0 (0.0)	0 (0.0)
Myeloma/lymphoma	1 (14.3)	16 (9.0)	0 (0.0)	14 (4.8)	1 (12.5)	17 (9.3)	1 (12.5)	14 (24.1)	2 (25.0)	3 (30.0)
Lung cancer	1 (14.3)	19 (10.7)	2 (22.2)	69 (23.5)	3 (37.5)	60 (32.8)	3 (37.5)	18 (31.0)	3 (37.5)	3 (30.0)
Other tumors	2 (28.6)	35 (19.7)	3 (33.3)	102 (34.8)	3 (37.5)	68 (37.2)	2 (25.0)	15 (25.9)	3 (37.5)	4 (40.0)
*p* = 0.789										
**Time developing motor deficits**										
1–7 days	2 (28.6)	59 (33.1)	4 (44.4)	109 (37.2)	2 (25.0)	64 (35.0)	2 (25.0)	15 (25.9)	4 (50.0)	7 (70.0)
8–14 days	3 (42.9)	81 (45.5)	0 (0.0)	86 (29.4)	4 (50.0)	24 (13.1)	0 (0.0)	6 (10.3)	0 (0.0)	0 (0.0)
>14 days	2 (28.6)	38 (21.3)	5 (55.6)	98 (33.4)	2 (25.0)	95 (51.9)	6 (75.0)	37 (63.8)	4 (50.0)	3 (30.0)
*p* = 0.503										
**Pre-radiotherapy walking ability**										
No	3 (42.9)	74 (41.6)	4 (44.4)	123 (42.0)	2 (25.0)	72 (39.3)	3 (37.5)	19 (32.8)	4 (50.0)	5 (50.0)
Yes	4 (57.1)	104 (58.4)	5 (55.6)	170 (58.0)	6 (75.0)	111 (60.7)	5 (62.5)	39 (67.2)	4 (50.0)	5 (50.0)
*p* = 0.873										
**Number of affected spinal segments**										
1–2	1 (14.3)	26 (14.6)	4 (44.4)	94 (32.1)	5 (62.5)	125 (68.3)	8 (100)	55 (94.8)	8 (100)	10 (100)
**≥3**	6 (85.7)	152 (85.4)	5 (55.6)	199 (67.9)	3 (37.5)	58 (31.7)	0 (0.0)	3 (5.2)	0 (0.0)	0 (0.0)
*p* = 0.654										
**ECOG performance score**										
1–2	2 (28.6)	68 (38.2)	4 (44.4)	119 (40.6)	4 (50.0)	88 (48.1)	4 (50.0)	30 (51.7)	4 (50.0)	3 (30.0)
3–4	5 (71.4)	110 (61.8)	5 (55.6)	174 (59.4)	4 (50.0)	95 (51.9)	4 (50.0)	28 (48.3)	4 (50.0)	7 (70.0)
*p* = 0.837										

The *p*-values were obtained with the Cochran–Mantel–Haenszel test.

**Table 3:** Local progression-free survival at 12, 18, and 24 months after radiotherapy (RT), stratified by propensity score quintiles.

	Quintile 1		Quintile 2		Quintile 3		Quintile 4		Quintile 5	
	Phase 2 (N = 7) *n* (%)	Control (N = 178) *n* (%)	Phase 2 (N = 9) *n* (%)	Control (N = 293) *n* (%)	Phase 2 (N = 8) *n* (%)	Control (N = 183) *n* (%)	Phase 2 (N = 8) *n* (%)	Control (N = 58) *n* (%)	Phase 2 (N = 8) *n* (%)	Control (N = 10) *n* (%)
**Local failure up to 12 months after RT**										
No	5 (71.4)	146 (82.0)	9 (100)	235 (80.2)	7 (87.5)	144 (78.7)	8 (100)	51 (87.9)	7 (87.5)	8 (80.0)
Yes	2 (28.6)	32 (18.0)	0 (0.0)	58 (19.8)	1 (12.5)	39 (21.3)	0 (0.0)	7 (12.1)	1 (12.5)	2 (20.0)
*p* = 0.198										
**Local failure up to 18 months RT**										
No	5 (71.4)	146 (82.0)	9 (100)	235 (80.2)	7 (87.5)	143 (78.1)	8 (100)	51 (87.9)	7 (87.5)	7 (70.0)
Yes	2 (28.6)	32 (18.0)	0 (0.0)	58 (19.8)	1 (12.5)	40 (21.9)	0 (0.0)	7 (12.1)	1 (12.5)	3 (30.0)
*p* = 0.139										
**Local failure up to 24 months after RT**										
No	5 (71.4)	145 (81.5)	9 (100)	233 (79.5)	7 (87.5)	142 (77.6)	8 (100)	50 (86.2)	7 (87.5)	7 (70.0)
Yes	2 (28.6)	33 (18.5)	0 (0.0)	60 (20.5)	1 (12.5)	41 (22.4)	0 (0.0)	8 (13.8)	1 (12.5)	3 (30.0)
*p* = 0.117										

The *p*-values were obtained with the Cochran–Mantel–Haenszel tests.

**Table 4:** Local control (freedom from an in-field recurrence following irradiation, assessed in 647 patients with improvement or no further progression of motor deficits) at 12, 18, and 24 months after radiotherapy (RT), stratified by propensity score quintiles.

	Quintile 1		Quintile 2		Quintile 3		Quintile 4		Quintile 5	
	Phase 2 (N = 5) *n* (%)	Control (N = 153) *n* (%)	Phase 2 (N = 9) *n* (%)	Control (N = 244) *n* (%)	Phase 2 (N = 8) *n* (%)	Control (N = 150) *n* (%)	Phase 2 (N = 8) *n* (%)	Control (N = 54) *n* (%)	Phase 2 (N = 8) *n* (%)	Control (N = 8) *n* (%)
**Local failure up to 12 months after RT**										
No	5 (100)	146 (95.4)	9 (100)	235 (96.3)	7 (87.5)	144 (96.0)	8 (100)	51 (94.4)	7 (87.5)	8 (100)
Yes	0 (0.0)	7 (4.6)	0 (0.0)	9 (3.7)	1 (12.5)	6 (4.0)	0 (0.0)	3 (5.6)	1 (12.5)	0 (0.0)
*p* = 0.855										
**Local failure up to 18 months after RT**										
No	5 (100)	146 (95.4)	9 (100)	235 (96.3)	7 (87.5)	143 (95.3)	8 (100)	51 (94.4)	7 (87.5)	7 (87.5)
Yes	0 (0.0)	7 (4.6)	0 (0.0)	9 (3.7)	1 (12.5)	7 (4.7)	0 (0.0)	3 (5.6)	1 (12.5)	1 (12.5)
*p* = 0.796										
**Local failure up to 24 months after RT**										
No	5 (100)	145 (94.8)	9 (100)	233 (95.5)	7 (87.5)	142 (94.7)	8 (100)	50 (92.6)	7 (87.5)	7 (87.5)
Yes	0 (0.0)	8 (5.2)	0 (0.0)	11 (4.5)	1 (12.5)	8 (5.3)	0 (0.0)	4 (7.4)	1 (12.5)	1 (12.5)
*p* = 0.655										

The *p*-values were obtained with the Cochran–Mantel–Haenszel tests.

**Table 5:** Overall survival at 12, 18, and 24 months after radiotherapy (RT), stratified by propensity score quintiles.

	Quintile 1		Quintile 2		Quintile 3		Quintile 4		Quintile 5	
	Phase 2 (N = 7) *n* (%)	Control (N = 178) *n* (%)	Phase 2 (N = 9) *n* (%)	Control (N = 293) *n* (%)	Phase 2 (N = 8) *n* (%)	Control (N = 183) *n* (%)	Phase 2 (N = 8) *n* (%)	Control (N = 58) *n* (%)	Phase 2 (N = 8) *n* (%)	Control (N = 10) *n* (%)
**Death up to 12 months after RT**										
No	2 (28.6)	83 (46.6)	2 (22.2)	101 (34.5)	5 (62.5)	75 (41.0)	3 (37.5)	29 (50.0)	2 (25.0)	6 (60.0)
Yes	5 (71.4)	95 (53.4)	7 (77.8)	192 (65.5)	3 (37.5)	108 (59.0)	5 (62.5)	29 (50.0)	6 (75.0)	4 (40.0)
*p* = 0.291										
**Death up to 18 months after RT**										
No	2 (28.6)	80 (44.9)	2 (22.2)	95 (32.4)	4 (50.0)	69 (37.7)	3 (37.5)	28 (48.3)	2 (25.0)	3 (30.0)
Yes	5 (71.4)	98 (55.1)	7 (77.8)	198 (67.6)	4 (50.0)	114 (62.3)	5 (62.5)	30 (51.7)	6 (75.0)	7 (70.0)
*p* = 0.476										
**Death up to 24 months after RT**										
No	2 (28.6)	76 (42.7)	2 (22.2)	93 (31.7)	4 (50.0)	64 (35.0)	2 (25.0)	27 (46.6)	2 (25.0)	8 (100)
Yes	5 (71.4)	102 (57.3)	7 (77.8)	200 (68.3)	4 (50.0)	119 (65.0)	6 (75.0)	31 (53.4)	6 (75.0)	0 (0.0)
*p* = 0.403										

The *p*-values were obtained with the Cochran–Mantel–Haenszel tests.

**Table 6:** Effect of irradiation on motor deficits and post-treatment ability to walk, stratified by propensity score quintiles.

	Quintile 1		Quintile 2		Quintile 3		Quintile 4		Quintile 5	
	Phase 2 (N = 7) *n* (%)	Control (N = 178) *n* (%)	Phase 2 (N = 9) *n* (%)	Control (N = 293) *n* (%)	Phase 2 (N = 8) *n* (%)	Control (N = 183) *n* (%)	Phase 2 (N = 8) *n* (%)	Control (N = 58) *n* (%)	Phase 2 (N = 8) *n* (%)	Control (N = 10) *n* (%)
**Effect of irradiation on motor function**										
Improvement	1 (14.3)	48 (27.0)	8 (88.9)	64 (21.8)	4 (50.0)	53 (29.0)	6 (75.0)	24 (41.4)	5 (62.5)	5 (50.0)
No further progression	4 (57.1)	105 (59.0)	1 (11.1)	80 (61.4)	4 (50.0)	97 (53.0)	2 (25.0)	30 (51.7)	3 (37.5)	3 (30.0)
Deterioration	2 (28.6)	25 (14.0)	0 (0.0)	49 (16.7)	0 (0.0)	33 (18.0)	0 (0.0)	4 (6.9)	0 (0.0)	2 (20.0)
*p* = 0.393										
**Post-treatment ability to walk**										
Unable to walk	3 (42.9)	65 (36.5)	0 (0.0)	116 (39.6)	2 (25.0)	59 (32.2)	0 (0.0)	11 (19.0)	2 (25.0)	2 (20.0)
Able to walk	4 (57.1)	113 (63.5)	9 (100)	177 (60.4)	6 (75.0)	124 (67.8)	8 (100)	47 (81.0)	6 (75.0)	8 (80.0)
*p* = 0.079										

The *p*-values were obtained with the Cochran–Mantel–Haenszel tests.

## Data Availability

Further information regarding the PRE-MODE trial is available at clinicaltrials.gov (identifier: NCT03070431). The original contributions presented in the present study are included in the article. Further inquiries can be directed to the corresponding author.

## Abbreviations

The following abbreviations are used in this manuscript:

ECOG	Eastern Cooperative Oncology Group
EQD2	Equivalent Dose in 2-Gy Fractions
LPFS	Local Progression-Free Survival
MSCC	Malignant Spinal Cord Compression
OS	Overall Survival
PRE-MODE	High-precision Radiotherapy of Motor Deficits Due to Metastatic Spinal Cord Compression
RT	Radiotherapy
